# Wellbeing as Capability: Findings in Hearing-Impaired Adolescents and Young Adults With a Hearing Aid or Cochlear Implant

**DOI:** 10.3389/fpsyg.2022.895868

**Published:** 2022-06-23

**Authors:** Wouter J. Rijke, Anneke M. Vermeulen, Christina Willeboer, Harry E. T. Knoors, Margreet C. Langereis, Gert Jan van der Wilt

**Affiliations:** ^1^Audiologisch Centrum, Royal Dutch Kentalis, Sint-Michielsgestel, Netherlands; ^2^Department for Health Evidence, Radboud University Medical Center, Nijmegen, Netherlands; ^3^Department of Research, Pento, Speech and Hearing Centers, Apeldoorn, Netherlands; ^4^Behavioural Science Institute, Radboud University, Nijmegen, Netherlands

**Keywords:** capability approach, conversion factors, functionings, hearing aids, hard-of-hearing

## Abstract

In the Western world, for deaf and hard-of-hearing children, hearing aids or cochlear implants are available to provide access to sound, with the overall goal of increasing their wellbeing. If and how this goal is achieved becomes increasingly multifarious when these children reach adolescence and young adulthood and start to participate in society in other ways. An approach to wellbeing that includes personal differences and the relative advantages and disadvantages that people have, is the capability approach, as developed by Nobel Prize laureate Amartya Sen. Capability is the set of real opportunities people have to do and be things they have reason to value. We interviewed 59 young people, aged 13 through 25, with cochlear implants (37) or hearing aids (22) to capture their capability. We found that their hearing devices enabled them to actively participate in a predominantly hearing society, with few differences between cochlear implant and hearing aid recipients. They did, however, report challenges associated with prejudices and expectations, and with feeling poorly understood, all of which appeared to impact their capability. Through the lens of capability, alleged differences between hearing aid and cochlear implant recipients began to fade. We discuss the implications for initiatives focused on the long-term support young recipients of hearing devices to meet their specific requirements over time.

## Introduction

Deaf and hard-of-hearing (DHH) children who participate in hearing societies, may experience significant challenges in social, emotional, and psychological areas (Antia et al., 2012; Kouwenberg et al., 2012; Wolters, 2013; Snoddon and Underwood, 2014). When these children enter puberty and adolescence, they start arranging their lives more to their own choosing, are able to reflect on choices, and participate independently in society, such as sports, jobs, school, and hobbies. These developmental tasks entail establishing self-governance and autonomy through peer group interactions. Hearing devices such as cochlear implants and hearing aids aim to facilitate this for DHH children. These devices, however, impact more than communication and perception of sounds, especially during adolescence. Identity formation, relational and sexual development, and the transition from primary to secondary education are some characteristics of this phase of life and are at risk for DHH young people when communication is hindered (Klimstra et al., 2010; Tolman and McClelland, 2011). Despite substantial auditory gain from hearing devices, enabling speech perception that facilitates spoken language acquisition and academic skills, DHH adolescents and young adults still appear disadvantaged in psychosocial areas compared to typical-hearing peers, such as self-perceived social acceptance, physical appearance, and self-worth (Marschark et al., 2007; van Gent, 2012; Wolters, 2013). Evaluation of hearing devices is currently mainly concerned with the functionality of the hearing devices, since it is conditional for any further effects. Corresponding measures such as speech perception and indicators for academic skills (such as vocabulary and working memory) are therefore an essential first step in evaluating hearing devices. But to further evaluate the impact of hearing loss and the value and limitations of hearing devices, we believe there should be an assessment of how hearing devices contribute to a person’s ability to lead a life of their own choosing, and what they require to achieve it.

To meet the developmental tasks in a hearing society, these young people with hearing impairments experience specific challenges due to the impairment and technical limitations of the devices. Although speech perception in case of moderate hearing impairment with the use of hearing aids has been found comparable to that of profoundly hearing-impaired adolescents and young adults who use cochlear implants, the impact of these devices is distinct. For young people with cochlear implants, the auditory gain of the device is significantly larger, which might increase device dependency. This might effect this ability to lead a life of their own choosing and their requirements.

The capability approach, developed by Nobel prize laureate Amartya Sen, is an approach to capture wellbeing (Sen, 1979). Capability is defined as the set of real freedoms people have to do and be what they have reason to value. A capability set emerges from the interaction between available resources, conversion factors, and achieved capabilities, called functionings (Robeyns, 2003). For example, a certain activity that might be of interest to a young adult with hearing aids is to meet with friends. By assessing capability, we would gather information about necessary resources, such as hearing aids, transport, and money. In addition to resources, he or she might need acceptance of friends, living in close proximity, permission from parents, and self-esteem. These personal, social, and environmental factors together are the conversion factors. Functionings are observable activities and states of being, such as playing a game, laughing, and communicating. Information on all these elements would reflect the young adult’s real freedom (i.e., capability) to meet with friends. An assessment of capability in DHH young people has an important role in identifying key factors in support for achieving their personal goals in societal participation to improve the impact of health care. This would require both an account of what these particular young people have reason to value (their interests on an individual level) and an analysis of their activities (functionings) and conditions (resources and conversion factors). We aimed to learn about the capability of young people who use cochlear implants or hearing aids by asking about their daily lives, what they strive for, and what they need to accomplish this.

## Materials and Methods

### Participants

We included 59 young people who received hearing aids (22) or cochlear implants (37). We selected participants with a minimal age of 12 years old and a maximum age of 25 years old. The characteristics of the participants are listed in Table 1, including available information on hearing loss and speech perception abilities. We only selected hearing aid users with at least 35 dB hearing loss at the better ear (pure-tone average at 1, 2, and 4 kHz). Hearing aid users received audiological care in a regional audiological center and resided in both urban and rural areas and attended mainstream or special educational settings. Cochlear implant users received audiological care in a cochlear implant center with a national function, also including residents from urban and rural areas that attended mainstream or special educational settings. We did not preselect for education level, gender, or other demographic characteristics. We excluded participants who could not be understood by the interviewer, for example, non-Dutch speaking participants and those with additional (cognitive) severe complex needs.

**Table 1:** Demographic characteristics of the respondents, distinguishing recipients of hearing aids, and cochlear implants.

Characteristic			Recipients of cochlear implant(s)	Recipients of hearing aid(s)
n			37	22
Age in years	M (SD)		17.3 (3.7)	17.3 (3.6)
	Range		12.6–25.0	12.8–24.1
Age of first aid in years, M (SD)			4.0 (3.6)	4.8 (2.8)
Gender	male		17	15
	female		20	7
Education	secondary special education		3	2
	secondary education		21	11
	secondary vocational education		9	6
	higher education		4	1
	university education		0	2
Hearing device	unilateral		13	0
	bilateral		21	22
	bimodal		3	
Hearing loss in dB (pure-tone average at 1, 2 and 4 kHz)	M (SD)		>85^*^	56.1 (12.8)
	Range			37–77
	Missing			4
Speech perception (% correctly repeated phonemes)	65 dB in quiet	M (SD)	93.6 (6.8)	94.5 (9.9)
	45 dB in quiet	Range	67–100	60–100
		Missing	0	3
		M (SD)	87.1 (10.3)	73.8 (22.2)
		Range	45–100	24–100
		Missing	4	3
Speech supported by sign language	Yes		6	1
	No		31	21
Interview setting	Online		13	5
	Face-to-face		24	17

* *Audiological inclusion criteria for cochlear implantation is > 85 dB hearing loss*.

### Data Collection

We used a qualitative design (i.e., interviews) to capture capability. During a period of 2 years (2019 and 2020), patients were invited via mail to participate prior to their annual fitting of their hearing device and follow-up evaluation of their development in their out-patient clinic. The interviews were conducted in a consulting room in the out-patient clinic. Due to COVID-19 restrictions, 18 interviews were conducted digitally with a videoconference app (whereby). One participant used a sign language interpreter, while three participants were accompanied by their parents. The interviewer (WR, male, late-twenties, and typical hearing) had a background in psychology, was trained and experienced in qualitative research, and had not met participants earlier. Participants were informed before participating, and the goal of the research was reiterated prior to the interview. Interviews lasted between 30 and 50 min.

To understand the nature and development of capability in DHH young people who use cochlear implants and/or hearing aids, we used a deductive qualitative approach. Through one-on-one interviews, we aimed to collect information on participants’ resources, conversion factors, functionings, and interests. We framed the interviews based on the methodology used by Alkire (2002). We started by asking participants to tell about their daily lives and asked them to elaborate on interests, conditions, and activities. We used mainly open questions to encourage input from participants, while using seven topics as a framework of conversation. These topics were based on Finnis’ basic goods: knowledge, life, play, esthetic experience, sociability, practical reasonableness, and transcendence (Finnis, 1980). The interview protocol can be found in Appendix A. Interviews were fully audio recorded and converted to intelligent (non-verbatim) transcripts.

### Analysis

We deducted elements from the capability approach using directive content analysis, a methodological orientation using an existing theory (Mayring, 2000). Resources were coded as the materials and means necessary to achieve valuable functionings. The environmental, personal, and social factors that influence resources and functionings were coded as conversion factors. Functionings represent what people do and are. When participants told us what they found important or interesting it was coded as “interest.” Codes could overlap, for instance when subjects talked about playing a sport they liked (both functioning and interest). To determine the interrater reliability, a random set of eight interviews were coded by two independent raters; the first author and a PhD-student from another department (MacPhail et al., 2016). Coding and analysis were computer assisted, using ATLAS.ti version 8 for Windows. The determined interrater reliability between the two raters of the codes was 0.81, which means a high number of quotes were identically coded.

### Ethical Considerations

The research ethics committee of the Radboud University Nijmegen Medical Center states that the abovementioned study (reference number of the study: 2017-3684) does not fall within the remit of the Medical Research Involving Human Subjects Act (WMO).

## Findings

We structured the quotes of the respondents according to themes based on the impact of hearing loss and the value and limitations of hearing devices: what respondents articulated they gained in opportunities by device use, what wishes or preferences might have been adapted to cope, what preconceptions of others they experienced in societal situations, and what disadvantages they might have experienced. Gained opportunities relate to the increase in capability, while experienced disadvantages might indicate obstructions in resources or conversion factors. Their experienced preconceptions of others are a social conversion factor that is especially relevant during puberty and adolescence. Their adapted preferences could portray a certain degree of freedom, as some functionings might not have been available to them.

The following will describe the capability of the respondents in two ways. First, we will present interview outcomes framed by the four themes: gained possibilities, adapted preferences, preconceptions of others, and experienced disadvantages. Per theme, we will attempt to portray the general findings, supported by specific quotes from respondents.

Then, we will present an overview of the functionings, resources, conversion factors, and interests of the respondents. On a group level, a cochlear implant or hearing aid was the most obvious distinguishing factor between participants, as depicted in Table 1. We will present outcomes from the interviews with available context, such as age, gender, and education.

### Structured Outcomes

#### Gained Opportunities

According to respondents, cochlear implants and hearing aids provide not only the ability to hear more and better (24-year-old female, one cochlear implant since 9 years of age), but also opportunities they feel they would not have had without it, such as their job (23-year-old female, two cochlear implants, first since 9 years of age). They feel they can communicate, but also have silence to relax (19-year-old female, one cochlear implant since 20 months old, and 20-year-old male, two hearing aids, first since 6 years of age).

Interviewer: “What is the biggest advantage of having a cochlear implant?”

“Well, sometimes I forget I’m deaf. That’s the greatest happiness I’ve had in my life. Some think you take away an identity, but for me it has given an identity. I could not have made it this far without it. I do not know what that would have been like, of course, but it’s been so nice for me. I get fair chances. For example, if you apply for a job, the chances of getting hired are a lot less if you are deaf. So, I do not report it either. And then I can show my skills during a job interview.” (18-year-old male wears one cochlear implant (since 12 months of age), student secondary vocational education).

“I used to listen music only rarely. Five years ago, a new hearing aid. They threw in a ComPilot [wireless accessory]. When music was played, the correct tones were played. That has greatly increased my ability to listen to music. For example, I listened to a lot of Acda and the Munnik, very easy music to listen to for lyrics. And singing along. Because I did not care much for melody and beats. So, I also really hated instrumental music, classical music. While now, I’ll just listen to it all. I really listen to a lot of music now. Now I listen to everything together.” (20-year-old male wears two hearing aids (first since 6 years of age), student university education).

#### Adapted Preferences

Respondents from all participating ages found ways to deal with previously difficult hearing-related situations. They adapted their desires, but also learned in which situations they could thrive. For example, cycling at the back of a group so that voices come toward them from the front, thus facilitating perception, or taking strategic positions in a room that enable speech reading. They also asked teammates or friends for assistance during sport activities. Furthermore, one of them switched from working in a noisy bakery to stocking shelves.

“Sometimes I feel like I really want to be hearing, but that feeling is fading more and more. Because in the past I was really like, ‘I would really like to be hearing’, but I did not know my own culture and what benefits and disadvantages we have. And now I’m less like I want to be hearing, I’m glad I’m deaf. Really a lot of benefits. Perfect sleep, you do not want to know.”—16-year-old female wears one cochlear implant (since 20 months of age), attends mainstream high school.

“I also find myself enjoying it more when we are playing a game, because I feel more involved than when we are having a conversation. Of course, I also like to have a conversation, but I find it more fun to play a game.” (18-year-old male wears one cochlear implant (since 5 years of age), attends mainstream high school).

“At first, I sat at the front of the class, but I did not like that. Now I sit at the back of the class, so that I can look into the class. Then I do not have to look around all the time to see where the sound is coming from. So, I have a little more of an overview of the class. Then I can pay more attention myself, because I do not have to look behind me.” (14-year-old female, wears two hearing aids (first since 6 years of age), attends mainstream high school).

#### Preconceptions of Others

Young DHH people shared experiences about living with cochlear implants and hearing aids, and the image and taboo hearing tools evoked. They said that everyone thinks in boxes, also in the deaf community; you are either with the deaf or the cochlear implants group.

“But I do not want to be put in a box. I am well aware that I am hearing impaired. One time, while going out, a hearing-impaired girl asked why I wasn’t with hearing-impaired friends. Very weird. ‘You pretend to be something you are not’, she said. Almost aggressive. I think that’s such a label. I do not need that.” (22-year-old male, wears two hearing aids (first since 4 years of age), student academic education).

Also, people might overestimate or not understand what hearing tools can provide. While they lead to opportunities, there is still a lack of knowledge and awareness in society, participants said.

Interviewer: “How do you notice the prejudices?”

“The possibilities… With a cochlear implant you can certainly hear better. But that’s the pitfall. With a cochlear implant you cannot necessarily keep track of everything. It does not solve everything. My deaf community says that because of cochlear implants there are fewer deaf people. They think that with cochlear implants people know better because they can hear better, but they do not [know better].” (24-year-old female, wears one cochlear implant (since 9 years of age), works as a nurse).

#### Experienced Disadvantages

There are certain disadvantages of living with hearing loss that came up more often than others. Hearing aids and cochlear implants are not waterproof, which can be problematic for sports (transpiration) or in the rain. Also, respondents said hearing through hearing technologies can be tiring, leading to headaches, or losing concentration. In addition, hearing assistive technologies enable hearing in otherwise challenging, adverse listening situations, though they are not always experienced as such. Respondents mentioned the inconvenience using these technologies in school, when they moved from one classroom to another with different teachers every hour. Others experienced technical malfunctioning or vulnerable hardware parts breaking. For these respondents, the proper use of hearing assistive technologies was difficult to realize, and assistive technologies were even considered a burden. One respondent said he believed he needed more motivation to achieve similar goals as typical-hearing people.

“But I have to persevere, because actually, as a hearing-impaired person, you simply need more motivation wanting to achieve the same as a good hearing person.” (21-year-old male wears two hearing aids (first since 2 years of age), student secondary vocational education).

“I really like listening to music. I also tried to make music myself, then with recorder lessons, but I just cannot do that with my hearing. Singing seems like a lot of fun, but I cannot do that either. And I’m not that much into painting or drawing.” (13-year-old female wears two cochlear implants (first since 2 years of age), attends mainstream high school).

Interviewer: “Did you use hearing assistive technologies in high school?”

“In the beginning, yes. But you notice that it was developed by hearing people. May sound weird. But hearing assistive equipment is very much… All you hear is the teacher. It’s like the teacher is yelling in your ear. At least that’s how I experience it. This is also the case with hearing aids, for example. When I am talking, it points at someone. That seems very useful, but in reality, it really is worthless. Because it does not do you any good.” (22-year-old male wears two hearing aids (first since 5 years of age), student secondary vocational education).

Interviewer: “What is the biggest disadvantage of having a cochlear implant?”

“Well, the hearing on batteries, I find that so annoying.” (19-year-old male wears two cochlear implants (first since 26 months of age), works as an electrician).

### Capability

The input from the respondents led to an overview of functionings, resources, conversion factors, and interests that fits the participated DHH young people (see Table 2). For this is a qualitative approach, the focus was to identify which resources, conversion factors, and functionings were essential for capability (we included all issues that were brought up).

**Table 2:** Participants’ input in terms of capability elements.

In general			
Functionings	Resources	Conversion factors	Interests
Explain to others, but also not disclosing deafness	Cochlear implant^**^	Forgetting devices (personal factor that influences the use of the hearing device)	Not being different
Being laughed at	Hearing aid	Changing preferences, accepting deafness more	Not being overestimated in their ability to hear when aided
Last to laugh at jokes	Hearing assistive technologies	Positive attitude, more motivation than typical-hearing peers	Not being ashamed for appearance cochlear implant^**^
Cycling at the back to increase sound perception^*^	Cords for sports	Maintenance, cleaning, vulnerability device and batteries	Being able to take phone calls in noise
Drive car		Prejudices, perceptions of others, taboo, people do not know how to communicate	Being able to lie on head with cochlear implant^**^
Sleep well		Appearance of cochlear implant^**^	Being able to turn off hearing device
			Not being labeled
			Consideration of others
**Work and school**			
Functionings	Resources	Conversion factors	Interests
Being (highly) educated, have job(s)	Resources associated with special education	Less energy	Being able to become anything when growing up^**^
No early shift after late shift at hospital^**^			
Sitting in front for lip reading^**^, sit in back for overview^*^			
Not meeting friends from school			
**Leisure time**			
Functionings	Resources	Conversion factors	Interests
Swimming together, swimming alone	Water case^**^	Not waterproof	Enjoying environmental sounds
Listening to music		Wireless connection with phone	Being able to play an instrument
Activities with organization for young deaf and hard-of-hearing people^*^		Rain makes hearing aids disfunction^*^	Playing games rather than conversations^**^
Go to the bar, go to festivals, cinema, parties		Music can be noise	Being able to participate in group conversations
Play sports (with and without hearing device)		Cochlear implant does not fit in horse cap^**^	
		Help from teammates	

* *Exclusively mentioned by hearing aid users*.

** *Exclusively mentioned by cochlear implant users*. The table is organized in columns (vertical), not rows (horizontal). Elements between columns are therefore not linked.

Despite differences in devices to remediate the effects of hearing loss (i.e., cochlear implants and hearing aids), the presented data tell a notably consistent story. With a few exceptions, young people who experienced hearing loss and the subsequent treatment and guidance describe similar daily activities (functionings), requisite resources, personal, social, and environmental conversion factors, and interests. Interviewees shared details about their daily lives, both hearing-related and otherwise. In most aspects of their lives, their hearing loss manifested. They went to school or had jobs, but they needed additional resources, such as hearing-assistive-technologies. They met with friends, but not always with friends from school for participants attending special education, as they lived further away. They went to bars, festivals, parties, and the cinema, but environmental noise complicated conversations. They played sports, but they relied on teammates for communication. They rode their bike, but rain or wind decreased sound perception. They expressed desires to being able to take phone calls in noise, which can be difficult. They enjoy time in silence (without their hearing devices) but would like to be able to participate in group conversations more easily. They listened to music but really enjoyed it with a direct input in their hearing device.

## Discussion

Three insights emerged from our study, which we will consider before discussing limitations, implications, and the conclusion.

Firstly, through the lens of capability, alleged differences between hearing aid and cochlear implant recipients began to fade. Previous studies evaluating daily lives of hearing aid and cochlear implant users observed varied results on activities and quality of life. In Sweden, researchers found similar functioning in daily situations between young hearing aid and cochlear implant users (Anmyr et al., 2011), though they did find differences regarding neck and shoulder pain, usage of aids and hearing problems in certain activities. A multi-center study by Huber et al. (2015) showed that the mental health of young cochlear implant users without additional disabilities was comparable to typical-hearing peers, while Castellanos et al. (2018) stated that long-term cochlear implant users are at risk for difficulties in psychosocial adjustment, depending on delays and deficits in language and executive functioning.

Secondly, quotes that were obtained only from either young people with hearing aids or cochlear implants were sparse, although worth discussing briefly. One hearing aid user mentioned a strategy for receiving information. Instead of sitting up close to her teacher, one girl preferred the overview she had sitting in the back of class, seeing who talked. Unique to cochlear implant users was how they coped with the external parts of the device (i.e., a microphone, speech processor, external antenna, and a magnet). They told how its appearance could result in shame or inconvenience, for example. One person strikingly illustrated how his sense of hearing depended on a device. “Hearing on batteries” was how he experienced dealing with it. Also, one cochlear implant user expressed her desire for more career opportunities. A past study did suggest that young cochlear implant users, although well integrated into the hearing world, had a significantly lower correspondence between career aspiration and actual occupation (Huber et al., 2008).

Thirdly, it seems that many challenges DHH young people encountered were not exclusively related to having difficulties hearing sounds, but rather to external perceptions and prejudices. They mentioned “not wanting to be different,” “not being labeled,” “being overestimated,” and “dealing with others’ perceptions.” And while hearing peers seemed to lack understanding, members of the Deaf community could be dismissive as well. These societal issues, related to acceptance and prejudices, are often raised by the Deaf community (Christiansen and Leigh, 2004). Ellington and Lim (2013) did report a lack of understanding by others that could lead to low self-esteem in DHH children. Respondents in the current study exclusively strived for the typical-hearing societal norm, living with the expectations and pressure. They expressed the feeling to need more motivation to get fair opportunities, as they were aware of the pitfall of listening with hearing devices, hearing more, but not everything. Providing and designing an inclusive society for people with disabilities is not a favor, but a duty established in the United Nations Convention on the rights of persons with disabilities (2006). In the Netherlands, much remains to be done in this area, especially for DHH young adults (Van Den Heuij et al., 2018).

### Limitations and Implications

We acknowledge the potential biases that accompany our study design. Our broad inclusion criteria resulted in a highly heterogeneous research sample with a broad variety of contexts and personal histories. Also, being interviewed through video from home (as 18 participants were) could have impacted communication, although it did not lead to substantive issues. The COVID-19 regulations also prevented including a reference group of typical-hearing peers and complicated collecting information on clinical context (e.g., speech perception), which could have provided more insights on participants interview outcomes. We therefore cannot attribute causality to hearing aids or cochlear implants and capability, nor did we intend to. How DHH adolescents view themselves heavily depends on their context (such as ethnicity and culture), making studies with these target groups difficult to compare and extrapolate (Byatt et al., 2021). However, the present results are significant in at least two major respects.

First, the subject of evaluation of young people with cochlear implants and hearing aids is often focused on clinical outcomes (Sparreboom et al., 2014; Cushing and Papsin, 2015), health-related quality of life (Dixon et al., 2020), and school performance (Punch and Hyde, 2005; Sarant et al., 2015). This is, to our knowledge, the first assessment of capability in this population, which led to insights on how young people with cochlear implants and hearing aids had remarkably similar capability outcomes. Their resources and conversion factors to lead valuable lives often coincided, as did their interests. Having the freedom to choose valuable functionings has been related to higher wellbeing in European citizens, while additionally reducing the importance of other factors such as health, friendship, and financial security (Steckermeier, 2021).

Secondly, as capability might not differ significantly between users of cochlear implants and hearing aids, the efforts to strengthen their capability might be combined too. Respondents from both groups seemed to desire more awareness about living with hearing loss in their personal environment, in addition to a more informed public perception of hearing devices. The capability approach is, more than anything, a normative framework born from the realm of justice. Therefore, programs and interventions addressing these societal action points have a distinct moral value and should be supported as such.

## Conclusion

Young DHH people who use either cochlear implants or hearing aids reported perceiving opportunities through the use of these hearing devices they would not have without them. Their hearing devices enabled them access to a predominately hearing society, in which they actively participated. Unfortunately, these young people explicitly express feelings of uncertainty and falling short when they compare themselves to typical-hearing peers. When application of hearing devices aims to improve wellbeing and to prevent psychosocial problems, monitoring the development of a stable identity in DHH young people is essential. In their own perspective, these young people advocate more awareness of and insights in hearing loss in the broader society. An important practical issue is the weakness and limitations of the hardware. For initiatives focused on supporting DHH young people, these results are of considerable interest. In addition, manufacturers of hearing devices and hearing assistive technologies can benefit from feedback from these users too.

Traditional wellbeing evaluations of health interventions are often top-down, summative assessments aimed to facilitate cost-effectiveness or patient satisfaction. In our view, the current study shows how a formative focus on the development of wellbeing in terms of capability can lead to clues for personalized care, societal action points, and conversation topics for anyone involved with DDH young people. These subjects blur the line between care and policy, between responsibility and justice.

## Data Availability Statement

The datasets presented in this article are not readily available because the interviews contain highly personal and sensitive information, which we do not have the permission to share beyond this manuscript. Requests to access the datasets should be directed to wouter.rijke@radboudumc.nl.

## Ethics Statement

The studies involving human participants were reviewed and approved by Medical Ethical Committee Radboudumc. Written informed consent to participate in this study was provided by the participants’ legal guardian/next of kin.

## Author Contributions

AV, ML, GW, CW, and WR contributed to conception and design of the study. WR collected and managed data and wrote the first draft of the manuscript. AV, ML, CW, and WR performed statistical analysis. HK and GW wrote sections of the manuscript. All authors contributed to the article and approved the submitted version.

## Funding

This research was financially supported by the Auditory-Communicative Programme Board, ZonMw.

## Conflict of Interest

The authors declare that the research was conducted in the absence of any commercial or financial relationships that could be construed as a potential conflict of interest.

## Publisher’s Note

All claims expressed in this article are solely those of the authors and do not necessarily represent those of their affiliated organizations, or those of the publisher, the editors and the reviewers. Any product that may be evaluated in this article, or claim that may be made by its manufacturer, is not guaranteed or endorsed by the publisher.
