# Supplementary material for: Wellbeing as Capability: Findings in Hearing-Impaired Adolescents and Young Adults With a Hearing Aid or Cochlear Implant
Source: Front Psychol. 2022 Jun 23;13:895868. doi: 10.3389/fpsyg.2022.895868 (PMC9261909; doi:10.3389/fpsyg.2022.895868)
Supplement: Supplementary file 1 [file Data_Sheet_1.PDF]

Appendix A. Interview Protocol

1 Introduction

The following questions were used to capture the capability of Deaf and Hard-of-Hearing (DHH) young people. These questions are based on the methodology used by Alkire (2002).

The questions were meant to start a conversation about the participants' daily lives, with the aim to collect information on participants' resources, conversion factors, functionings, and interests. We used mainly open questions to encourage input from participants, while using seven topics as a framework of conversation. These topics were based on Finnis' basic goods: knowledge, life, play, aesthetic experience, sociability, practical reasonableness, and transcendence (Finnis 1980).

After the initial question, follow-up questions such as *"Can you elaborate on that?"*, *"What makes that you can/cannot do/be that?"*, and *"How did that happen?"* were essential to let participants articulate their own answers.

2 Questions

Can you tell me how a typical day in a typical week looks for you? What do you do, where do you go?

Can you tell me about your leisure time? (sports, hobbies, clubs)

Can you tell me about your experiences in school?

Can you tell me about your (side) job?

How do you communicate with friends, government agencies, health care institutions?

Can you tell me about resources or tools aside from your hearing device that you rely on?

Can you tell me about your social life? Does it include hearing and DHH people?

What do you do when you need support, social or otherwise?

What do you like/dislike about your hearing device?

Are there things you would like to be able to do, but are not able to (yet)?

(if appropriate:) What has changed since you received your hearing device?

3 References

Alkire, S. (2002). Valuing freedoms: Sen's capability approach and poverty reduction. Oxford ; New York, Oxford University Press.

Finnis, J. (1980). Natural Law and Natural Rights, Oxford University Press UK.
